# Modulation of L-Type Calcium Currents by Resveratrol-Induced Myogenesis in C2C12 Cells

**DOI:** 10.3390/cells15070650

**Published:** 2026-04-06

**Authors:** Andrea Biagini, Luana Sallicandro, Jasmine Covarelli, Rosaria Gentile, Alessandra Mirarchi, Alessio Farinelli, Gianmarco Reali, Diletta Del Bianco, Paola Tiziana Quellari, Elko Gliozheni, Antonio Malvasi, Giorgio Maria Baldini, Giuseppe Trojano, Claudia Tubaro, Claudia Bearzi, Roberto Rizzi, Cataldo Arcuri, Paolo Prontera, Andrea Tinelli, Bernard Fioretti

**Affiliations:** 1 Department of Chemistry, Biology and Biotechnologies, University of Perugia, Via dell’Elce di Sotto 8, 06132 Perugia, PG, Italy; andrea.biagini@dottorandi.unipg.it (A.B.); luana.sallicandro@dottorandi.unipg.it (L.S.); rosaria.gentile@dottorandi.unipg.it (R.G.); alessandra.mirarchi@unipg.it (A.M.); alessio.farinelli@unipg.it (A.F.); gianmarco.reali@dottorandi.unipg.it (G.R.); diletta.delbianco@dottorandi.unipg.it (D.D.B.); paolatiziana.quellari@dottorandi.unipg.it (P.T.Q.); elkogliozheni@dottorandi.unipg.it (E.G.); claudiatubaroct@gmail.com (C.T.); 2 Department of Medicine and Surgery, University of Perugia, Piazza Lucio Severi 1, 06132 Perugia, PG, Italy; jasmine.covarelli@dottorandi.unipg.it (J.C.); cataldo.arcuri@unipg.it (C.A.); paolo.prontera@ospedale.perugia.it (P.P.); 3 ASST Grande Ospedale Metropolitano Niguarda, 20162 Milano, Italy; 4 Department of Obstetrics and Gynecology, Faculty of Medicine, University of Tirana, AL1005 Tirana, TR, Albania; 5 Department of Biomedical Sciences and Human Oncology, University of Bari, 70121 Bari, BA, Italy; antoniomalvasi@gmail.com; 6 MOMO Ferti LIFE IVF Center, 76011 Bisceglie, Italy; gbaldini97@gmail.com; 7 Department of Maternal and Child Health, “Madonna delle Grazie” Hospital ASM, 75100 Matera, MT, Italy; giuseppe.trojano@asmbasilicata.it; 8 National Research Council-Institute for Biomedical Technologies (CNR-ITB), 20054 Segrate, MI, Italy; claudia.bearzi@cnr.it; 9 Department of Well-Being, Health and Environmental Sustainability (BeSSA), Sapienza University of Rome, 02100 Rieti, RI, Italy; roberto.rizzi@uniroma1.it; 10 Department of Obstetrics and Gynecology and CERICSAL (CEntro di RIcerca Clinico SALentino), Veris delli Ponti Hospital, Via Giuseppina delli Ponti, 73020 Scorrano, LE, Italy

**Keywords:** resveratrol, calcium channel, potassium channel, myocyte, myogenesis

## Abstract

Skeletal muscle differentiation is tightly regulated by membrane potential dynamics and voltage-dependent ion channel activity. Potassium (K^+^) and calcium (Ca^2+^) currents cooperate to orchestrate the transition of myoblasts into fusion-competent myotubes, and alterations in this process are associated with dystrophic phenotypes. Here, we investigated the electrophysiological remodeling accompanying C2C12 myogenesis and the modulatory effects of the polyphenol resveratrol (RES) on calcium voltage-gated channel subunit alpha 1 S (CACNA1S, Cav1.1, L-type) currents. Whole-cell patch-clamp recordings were performed in proliferating and differentiating C2C12 cells to characterize the temporal expression of K^+^ currents and voltage-dependent Ca^2+^ channels (VDCCs). During differentiation, three electrophysiological subpopulations were identified according to K^+^ current profiles: SK4+/EAG−/Kir−, SK4−/EAG+/Kir−, and SK4−/EAG+/Kir+. This sequence paralleled a progressive membrane hyperpolarization from −20 mV to −70 mV, consistent with the physiological maturation of myogenic cells. In C2C12 myocytes, nimodipine-sensitive L-type currents were the only Ca^2+^ conductance observed. Their activation threshold (~−30 mV) and half-activation voltage (V/2 ≈ −12 mV) indicated the co-expression of embryonic and adult Cav1.1 isoforms. Exposure to RES (30 µM, 48 h) produced a depolarizing shift in activation (ΔV/2 ≈ +9 mV) and a reduction in current amplitude across all voltages, consistent with a transition toward the adult splice variant of Cav1.1. These findings suggest that RES promotes electrophysiological maturation of skeletal muscle cells by modulating calcium channel expression and gating behavior. Given its known ability to correct splicing abnormalities in *CACNA1S* and related genes, resveratrol emerges as a promising pharmacological agent for restoring calcium homeostasis in neuromuscular disorders such as myotonic dystrophy type 1 (DM1).

## 1. Introduction

Human competent myoblast fusion is mediated by an increase in cytosolic Ca^2+^ level that results from Ca^2+^ influx through low-threshold voltage-activated (LVA) Ca channels. These LVA-sustained “window” Ca^2+^ currents are present within a narrow range of membrane potentials and are the result of their combined steady-state activation and inactivation properties [1]. By controlling membrane potential and in turn the LVA-sustained “window” Ca^2+^ currents, other ion currents modulate the myoblast fusion process [1,2,3]. Membrane potential and ion channel activity are major determinants in calcium entry and intracellular calcium signaling in skeletal muscle cells [4]; the main currents involved in membrane potential regulation of competent human myoblasts are the following K^+^ currents: hEAG (human ether-à-go-go) [1], Kir (inward rectifier K^+^ currents) [5], and HERG (human ether-à-go-go-related gene) [2]. The expression of these ion currents is strictly associated with myogenic programs of currents that appear following a precise sequence: hEAG, Kir, HERG, and before fusion, LVA Ca^2+^ current; perturbation of this sequence can abolish myoblast fusion [6,7]. The C2C12 myocytes over-expressing hERG increases intracellular Ca^2+^ through modulation of calsequestrin-1, ryanodine receptor 1 (RYR1)-related signaling, excitation-coupled calcium entry and store-operated Ca^2+^ entry [8].

Resveratrol (3,5,4′-trihydroxy-trans-stilbene, RES), a natural polyphenol with stilbenic structure found in red wine, has received increasing attention for the ability to prevent or slow the progression of a variety of pathologies [9]. Epidemiologic studies suggest that consumption of mild-to-moderate amounts of red wine may reduce the incidence of coronary heart disease [10,11], and RES is thought to be responsible [10,12]. The molecular basis for the bioactivity of this compound remains unclear; however, it has been reported to confer cardioprotective effects including antioxidant [13] and anti-inflammatory activity [14], preconditioning against ischemic injury [15], reduced ischemia–reperfusion injury and infarction [16], attenuated hypertrophic response [17,18], enhanced peri-infarct neovascularization [19], and antiarrhythmic efficacy [20,21]. Furthermore, it has been shown that RES directly inhibits cardiac fibroblast proliferation and differentiation in vitro [22,23], essential features of fibrosis and structural remodeling. These latter cardioprotective effects attributed to RES support the premise that it may represent a potentially beneficial therapeutic agent to ameliorate cardiac function and remodeling following an ischemic insult. In addition, RES has been shown to modulate multiple intracellular signaling pathways involved in cellular metabolism, inflammation, and survival [24]. The RES mechanism of action has not been completely defined yet, and several targets directly and indirectly have been proposed for explaining its cellular effects: (1) the activation of a sirtuin class of nicotinamide adenine dinucleotide (NAD^+^)- dependent deacetylases; (2) the modulation of adenosine monophosphate (AMP)-activated kinase (AMPK); (3) the inhibition of type IV phosphodiesterase (PDE4). Interestingly, RES may exert a cardioprotective action dependent on adenosine triphosphate (ATP)-sensitive potassium channel (K_ATP_) activity [25], which could indicate that the activation of K^+^ currents and the resulting hyperpolarization induced by RES represent the mechanism promoting myogenesis [26]. Moreover, modulation of AMPK signaling has emerged as a relevant mechanism in myotonic dystrophy type 1 (DM1), where pharmacological and physiological activation of AMPK improves the spliceopathy in skeletal muscle [27].

Voltage-dependent calcium channels (VDCCs) play a crucial role in skeletal muscle excitation–contraction coupling, and their expression and functional properties undergo developmental modulation [28,29,30]. In pathological conditions such as muscular dystrophies, myotubes often revert to an embryonic phenotype, displaying altered calcium handling and expression of immature isoforms of calcium channel subunits [31,32,33,34]. Consistently, altered functional differentiation and calcium-handling properties have also been described in progenitor cells from genetic myopathy models, supporting the idea that ion-channel remodeling and Ca^2+^ dysregulation are integral components of the diseased muscle phenotype [24]. In this context, the identification of pharmacological agents capable of modulating calcium homeostasis and correcting molecular abnormalities associated with dystrophic myotube phenotypes is of growing interest. Notably, RES is able to modulate gene expression and alternative splicing, possibly through direct interaction with nucleic acids [35]. In various neuromuscular disorders, including spinal muscular atrophy (SMA), RES has been reported to enhance exon 7 inclusion of the survival motor neuron 2 (*SMN2*) pre-mRNA in cellular models [36,37], and to rescue aberrant splicing in the alpha glucosidase (*GAA*) gene in patient-derived fibroblasts affected by acid maltase deficiency [38]. More broadly, DM1 muscle dysfunction is currently viewed as the result of multiple converging mechanisms, including RNA toxicity, spliceopathy, altered signaling pathways, muscle wasting, and calcium dysregulation [33]. In DM1 it was demonstrated that RES can correct the splicing defect of the insulin receptor (*INSR*) pre-mRNA in DM1 fibroblasts, suggesting its potential as a therapeutic agent [39]. Importantly, muscle weakness in DM1 has been associated with dysregulated splicing and altered gating of the calcium voltage-gated channel subunit alpha 1 S (*CACNA1S*, Cav1.1, L-type) channel, further linking splice defects to abnormal calcium handling in skeletal muscle [34]. Moreover, since skeletal muscle is among the most compromised tissues in DM1, RES has been evaluated in myotubes derived from DM1 patients, where it has been shown to correct aberrant splicing of *RYR1* pre-mRNA and improve Ca^2+^ signaling in DM1 myotubes [40]. Given these findings, we propose investigating the effects of RES on voltage-dependent calcium currents in the C2C12 myocyte cell line, characterized by the expression of embryonic-type calcium channels.

## 2. Materials and Methods

### 2.1. Cell Culture

The murine skeletal myoblast cell line C2C12 (at passages 15–30) was obtained from the American Type Culture Collection (ATCC, Rockville, MD, USA; CRL 1772). C2C12 proliferating myoblasts, plated at 2000 cells/cm^2^, were maintained in a humidified atmosphere (CO_2_ 5%-air 95%, 37 °C) and passaged by standard trypsinization every 3 days in a growth medium (GM) consisting of DMEM supplemented with 20% fetal bovine serum, 4 mM L-glutamine, 100 IU/mL penicillin, and 100 μg/mL streptomycin (GIBCO BRL, Gaithersburg, MD, USA). For differentiation-committed cells, cells were plated at 6000/cm^2^ directly in a differentiation medium (DM) consisting of DMEM supplemented with 2% horse serum (HS), 4 mM L-glutamine, 100 IU/mL penicillin, and 100 μg/mL streptomycin (GIBCO). The experiments were carried out both on proliferating myoblasts at days 1–4 after being plated in either GM and on differentiation-committed cells (myocytes) at days 1–4 after plating in DM or DM plus 30 μM resveratrol. For quantitative RT-PCR experiments, cells were plated at 70,000 cells per 35 mm dish and treated with 5 μM resveratrol. A lower concentration was used in these experiments to minimize the antiproliferative effects of resveratrol, whereas electrophysiological recordings were performed in the presence of 30 μM resveratrol. All reagents were purchased from Euroclone S.p.A. (Pero, MI, Italy).

### 2.2. Electrophysiology, Solutions, and Drugs

Whole-cell-perforated patch-clamp recordings were performed on C2C12 myoblasts using amphotericin B to gain intracellular access. Electrode resistance ranged from 3 to 5 MΩ. Currents were amplified using a HEKA EPC-10 amplifier and analyzed with PatchMaster (HEKA Elektronik GmbH, Reutlingen, Germany) and Origin 6.1 software. For online data acquisition, signals were filtered at 3 kHz and sampled every 40 μs. Membrane capacitance (Cm) was measured using the “Membrane Test” routine provided in the software. Recordings were performed both on proliferating myoblasts and differentiation-committed cells cultured in 35 mm Petri dishes (Falcon, Corning, Glendale, AZ, USA), as described in Fioretti et al. [41]. To study early differentiation events, low-density cultures were maintained in fusion-promoting medium, allowing cells to initiate differentiation without fusing, remaining mononucleated due to lack of cell–cell contact. Macroscopic potassium currents were recorded in whole-cell perforated configuration [41,42,43]. The extracellular (bath) solution—Physiological Salt Solution (PSS)—contained (in mM): 106.5 NaCl, 5 KCl, 2 CaCl_2_, 2 MgCl_2_, 5 MOPS, 20 glucose, and 30 Na-gluconate; pH was adjusted to 7.25. The pipette solution contained (in mM): 57.5 K_2_SO_4_, 55 KCl, 5 MgCl_2_, and 10 MOPS. All chemicals were of analytical grade. Ionomycin was prepared as a stock solution in DMSO. For the recording of voltage-activated L calcium current components, protocols described in Fioretti et al. [44] were followed. The internal pipette solution contained (in mM): 100 Cs-methanesulfonate, 30 HEPES, 1 MgCl_2_, 10 EGTA, 8 CsCl, 4 MgATP, 0.5 NaGTP, and 1 cAMP; pH was adjusted to 7.4 with CsOH. The initial seal formation and cell break-in were performed in external Tyrode solution. Subsequently, cells were perfused with a solution for calcium current recording containing (in mM): 147 tetraethylammonium chloride (TEA-Cl), 2 CaCl_2_, 10 HEPES, 10 glucose, and 5 μM nimodipine; pH 7.4 was adjusted with TEA-OH.

### 2.3. RNA Isolation, Reverse Transcription, and Quantitative RT-PCR (qRT-PCR) of C2C12 Cells

Total RNA was extracted from C2C12 pellets using TRIzol reagent (Thermo Fisher Scientific, Rodano, MI, Italy, Cat. No. 15596026) according to the manufacturer’s instructions. For each sample, 1 µg of RNA was reverse transcribed using the SensiFAST cDNA Synthesis Kit (Meridian Bioscience, Villa Cortese, MI, Italy, Cat. No. BIO-65053) following the manufacturer’s protocol. qRT-PCR was performed using gene-specific primers for target genes: *Myod1* (NM_010866.2), *Myog* (NM_031189.2), and *Cacna1s* exon 29 (NM_001081023.3). Reactions were carried out using SYBR Green fluorescent dye (SensiFAST SYBR Lo-ROX Kit, Meridian Bioscience, Cat. No. BIO-94005). Amplifications were performed on a QuantStudio™ 1 Real-Time PCR System (Applied Biosystems, Thermo Fisher Scientific) using standard cycling conditions recommended by the manufacturer. Mouse glyceraldehyde-3-phosphate dehydrogenase (*Gapdh)* was used as the normalizing control in all qRT-PCR assays. Relative transcript levels were calculated using the comparative Ct (ΔΔCt) method [45].

For the real-time PCR, the primer sequences were:
*Myod1*_FWAAGACGACTCTCACGGCTTG*Myod1*_RVGCAGGTCTGGTGAGTCGAAA*Myog*_FWCAGCCCAGCGAGGGAATTTA*Myog*_RVAGGCTTTGGAACCGGATAGC*Cacna1s*_*exon29*_FWATCGTCATCGGCAGCATCAT*Cacna1s*_*exon29*_RVCAGCAGCTTGACCAGTCTCA*Gapdh*_FWCATCACTGCCACCCAGAAGACTG*Gapdh*_RVATGCCAGTGAGCTTCCCGTTCAG

### 2.4. Murine Fibroblast Reprogramming

Reprogramming was performed as previously described [46]. Briefly, mouse fibroblasts were reprogrammed into induced pluripotent stem cells (iPSCs) using lentiviral vectors expressing pluripotency-associated transcription factors. Following viral transduction, cells were maintained under standard culture conditions for four days. Cells were then trypsinized and replated onto a feeder layer of mitotically inactivated mouse embryonic fibroblasts (MEFs) and cultured in pluripotency maintenance medium. Approximately 15–18 days after transduction, colonies displaying typical pluripotent stem cell morphology were identified, manually picked, and expanded under feeder-dependent culture conditions for further experiments.

### 2.5. Murine iPSC Differentiation and Pharmacological Treatment

Differentiation of iPSCs toward the cardiomyocyte lineage was performed according to previously published protocols [46]. Briefly, undifferentiated iPSCs were first exposed to bone morphogenetic protein-2 (BMP-2) for 12 h, then harvested by trypsinization and resuspended in embryoid body differentiation medium (EBD medium). A total of 8 × 10^5^ cells were distributed as 800 cells per drop to induce cardiac differentiation and generate embryoid bodies (EBs) using the hanging-drop method. Resveratrol (RES; Cat. #R5010, Sigma-Aldrich, Merck, Darmstadt, Germany) stock solution was prepared in dimethyl sulfoxide (DMSO) at 30 mM and diluted in EBD medium to obtain a final concentration of 15 μM. On day 3 of differentiation, EBs were transferred from hanging drops to 6-well low-adhesion plates containing fresh EBD medium supplemented with the appropriate treatment. On day 5, floating EBs were transferred to gelatin-coated culture plates and maintained in EBD medium under different experimental conditions. Treatments were renewed every other day by replacing the medium. On day 12 of differentiation, cells were harvested in Trizol reagent and stored at −80 °C for subsequent RNA extraction and molecular analyses.

### 2.6. Gene Expression Profiling for Murine iPSC

Expression of genes was analyzed by qRT-PCR. Total RNA was extracted from cells under different conditions using Trizol (Invitrogen). 1 g of total RNA was reverse transcribed to cDNA using SuperScript III Kit (Invitrogen) to be amplified by qRT-PCR. Syber Green PCR master mix (Applied Biosystems) and specific primers for cardiac markers of differentiation and for pluripotency were used, as below. Each sample was analyzed in triplicate using the Tetrad 2 (Biorad) and 7900HT qRT-PCR (Applied Biosystems). For the qRT-PCR analysis of CM-iPSCs, the following primers were used:
**Gene**
**qRT-PCR Primer Set*****GAPDH***forwardGGCAAATTCAACGGCACA
reverseGTTAGTGGGGTCTCGCTCTG***Oct4***forwardCCCTCTGTTCCCGTCACTG
reverseACCTCCCTTGCCTTGGCT***Brachyury***forwardCAGCCCACCTACTGGCTCTA
reverseGAGCCTGGGGTGATGGTA***GATA4***forwardTCTCACTATGGGCACAGCAG
reverseGCGATGTCTGAGTGACAGGA***NKX2.5***forwardCAAGTGCTCTCCTGCTTTCC
reverseGGCTTTGTCCAGCTCCACT***TBX5***forwardCGAAGTGGGCACAGAGATG
reverseCACCTTCACTTTGTAACTAGGAAACA***α-MHC***forwardCGCATCAAGGAGCTCACC
reverseCCTGCAGCCGCATTAAGT***β-MHC***forwardCGCATCAAGGAGCTCACC
reverseCTGCAGCCGCAGTAGGTT***TNNI***forwardGCAGGTGAAGAAGGAGGACA
reverseCGATATTCTTGCGCCAGTC


### 2.7. Statistical Analysis

All data are reported as the mean ± standard error (SE). Student’s *t*-test and one-way ANOVA were used to evaluate differences between groups, and *p* < 0.05 was considered statistically significant. Statistical analyses were performed with Origin 6.1 software (OriginLab Corporation, Northampton, MA, USA).

## 3. Results

### 3.1. Potassium Currents Profile During Myoblast Differentiation

**Figure 1:**
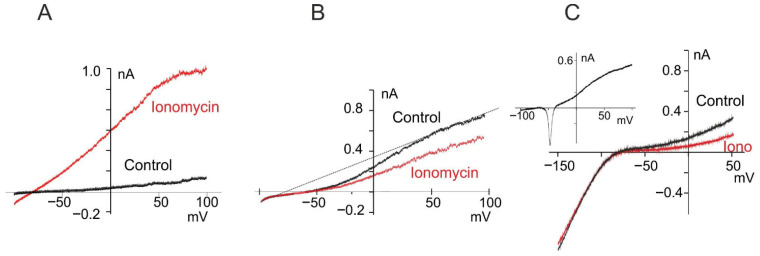
Electrophysiological profile of C2C12 myoblast subpopulations during myogenic differentiation. (**A**) Current–voltage (I–V) relationship recorded from a proliferating C2C12 myoblast in growth medium, using voltage ramps from −100 mV to +100 mV (holding potential: 0 mV), under control conditions (black) and following ionomycin application (500 nM, red). (**B**) I–V relationship from a myoblast cultured for 4 days in differentiation medium, recorded using the same protocol as in A. The dashed line intersects the control trace at approximately −88 mV—a value close to the calculated potassium equilibrium potential (−90 mV, from the Nernst equation)—indicating the potassium-selective nature of the voltage-dependent current. The red trace (post-ionomycin) shows calcium-induced inhibition of the voltage-activated current, a hallmark feature of EAG channel activity. (**C**) A subset of EAG-positive myoblasts exhibited an additional Kir current, characterized by increased conductance at highly negative potentials. In rare cases, the presence of voltage-activated sodium currents was also detected (**inset**). For Kir characterization, voltage ramps started from −150 mV (V_h_ = 0 mV).

In order to assess the myogenesis process from myoblasts to myocytes, we evaluated the potassium current profile as a differentiation marker. The expression of potassium currents associated with the myogenic program in C2C12 cells was analyzed using voltage ramps ranging from −100 to +100 mV from a holding potential (Vh) of 0 mV to obtain I–V relationships. In 14 proliferating myoblasts, a linear I-V within the full range of potentials was observed (Figure 1A, black trace). In accordance with previous studies, application of calcium ionophore to bath solution increases the conductance at each potential explored, with the exception of values near E_K_ (Figure 1A, red trace) associated with SK4 expression in proliferating myoblasts [41]. Following 4-day growth in the differentiating medium, a delayed rectifying potassium (DRK) current appears (*n* = 6, Figure 1B, black trace), which has properties similar to those previously described by our group [41]. Interestingly, following the addition of ionomycin, no current ascribed to SK4 channels was observed, and the DRK current was inhibited (Figure 1B, red trace). These data are in accordance with the calcium inhibition of EAG currents, as described in human myoblasts during the early phase of myogenesis [1]. Myoblasts that express DRK could also express other currents. In some cases (2/6), an inward Na^+^ current was recorded (inset Figure 1C, this current was not studied further), as well as the classic signature of an inward rectifier potassium current (*n* = 3, Figure 1C), similar to that expressed in human myoblasts in the late phase of differentiation [5].

**Figure 2:**
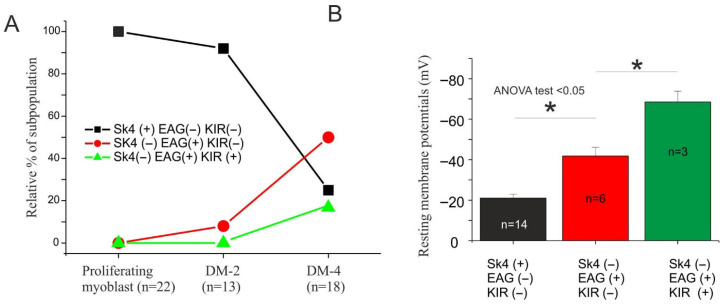
C2C12 myoblast hyperpolarization during myogenesis is driven by differential expression of potassium currents. (**A**) Relative distribution (%) of three electrophysiologically distinct subpopulations of C2C12 myoblasts at different stages of myogenic differentiation: proliferating myoblasts (growth medium), and myoblasts after 2 and 4 days in differentiation medium (DM-2 and DM-4). Subpopulations are classified based on potassium current profiles: SK4+/EAG−/Kir− (black squares); SK4−/EAG+/Kir− (red circles); SK4−/EAG+/Kir+ (green triangles). Proliferating myoblasts predominantly express SK4 currents, whereas differentiation is associated with a progressive increase in EAG+ and Kir+ subpopulations, indicating a functional remodeling of potassium channel expression during myogenesis. (**B**) Resting membrane potential (RMP) measurements of the same electrophysiological subtypes shown in A. SK4+ cells exhibit a depolarized RMP (−21.0 ± 1.9 mV, *n* = 14), EAG+ cells show intermediate hyperpolarization (−41.8 ± 4.2 mV, *n* = 6), while Kir+ myoblasts display the most negative RMP (−68.5 ± 5.3 mV, *n* = 3), consistent with the contribution of Kir currents. Statistical analysis (one-way ANOVA, *p* < 0.05) indicates significant differences between all groups (* *p* < 0.05).

Three major subpopulations can be identified based on the potassium current expression: (a) SK4+/EAG−/Kir− that begins to disappear at day 2 of differentiation; (b) SK4−/EAG+/Kir− that starts to appear at day 2; (c) SK4−/EAG+/Kir+ that begins to appear at day 4 of differentiation (Figure 2A). These electrophysiologic events are compatible with the sequential up- and down-regulation of ion channels during myogenesis, similar to that observed in human myoblast models [3,47]. A dedicated experiment was performed to evaluate the resting membrane potentials of these subpopulations at different differentiation stages (Figure 2B). Proliferating myoblasts (SK4+/EAG−/Kir−) display more depolarizing membrane potential (−21.1 ± 2 mV, *n* = 14), whereas (SK4−/EAG+/Kir+) subpopulation displays a more hyperpolarizing value (−68.5 ± 5 mV, *n* = 3). The intermediate subpopulation (SK4−/EAG+/Kir−) displays an intermediate hyperpolarizing value (−41.8 ± 4.2 mV, *n* = 6). Altogether these preliminary results show that the myogenic mouse cell line C2C12, during differentiation, hyperpolarizes the membrane potential as a consequence of differential potassium current expression, in accordance with what observed in murine and human myoblasts derived from freshly isolated satellite cells [3,6,7].

### 3.2. Calcium Currents Expression in C2C12 Myocytes

**Figure 3:**
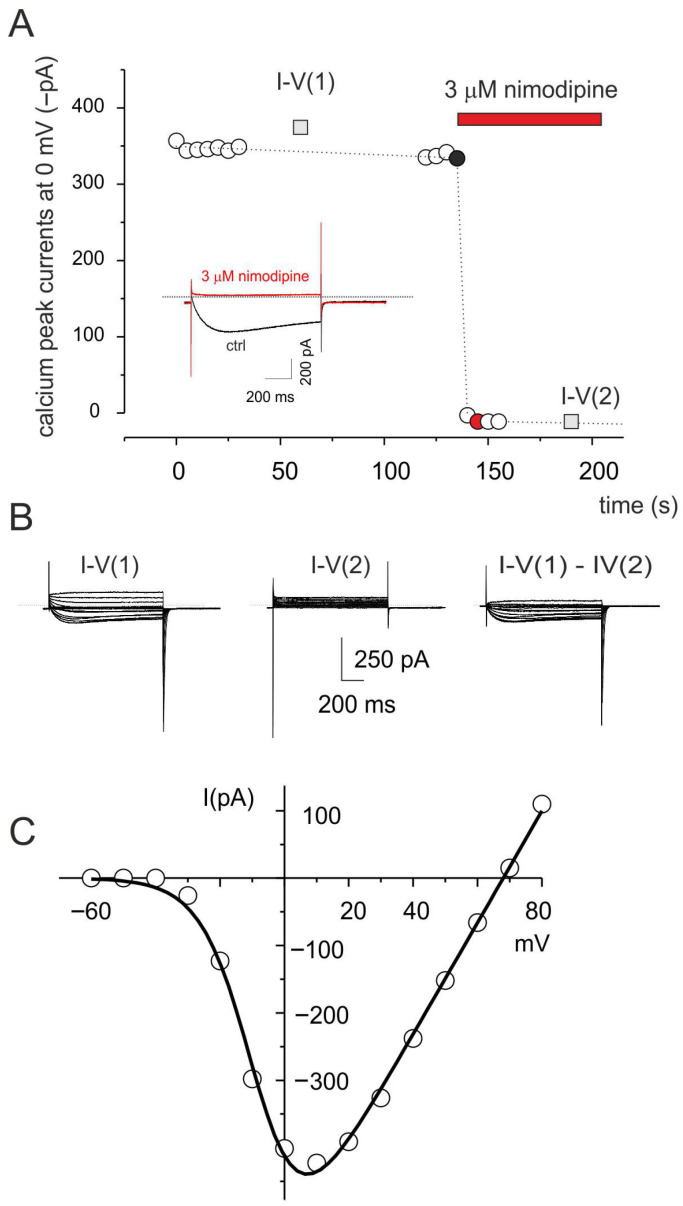
L-type calcium currents in C2C12 myocytes. (**A**) Time course of peak whole-cell Ca^2+^ current recorded at 0 mV (every 5 s from a holding potential of −60 mV) during a representative experiment designed to isolate L-type Ca^2+^ current components. The red bar indicates the period of nimodipine application. The gray squares represent the calcium peak currents at 0 mV obtained from the labeled I–V voltage-step protocol (from −60 mV to +80 mV, 1000 ms steps) before and after nimodipine application. The black circle corresponds to the time point at which the control trace was recorded, whereas the red circle corresponds to the time point at which the trace in presence of nimodipine was recorded. Inset (**left**): representative current traces recorded under control conditions (black), in the presence of nimodipine (red) at 0 mV. (**B**) Families of Ca^2+^ current traces evoked by depolarizing steps from −60 mV to +80 mV (holding potential −60 mV), recorded before (IV-1, left) and after (IV-2, middle) nimodipine application, whereas on the right is the L-type calcium currents obtained by digital subtraction of current families I-V(1) with IV(2). (**C**) I–V relationship of peak L-type Ca^2+^ currents plotted against membrane voltage, derived from the digital subtraction of current family traces. Dotted lines represent fits to the Boltzmann-modified equation: I = G_max_·(V − V_rev_)/(1 + exp[(V/2 − V)/k]), with best-fit parameters: V/2 = −12.0 mV, k = 5.6 mV.

Competent C2C12 myoblasts ready to differentiate into myocytes express high voltage L-type calcium current ascribed to L-type calcium channel (Cav1.1 subunit), in line with other muscle preparations [48]. A typical calcium current recording isolated from a C2C12 myocyte, representative of five recordings, is shown in Figure 3A, where the dots represent the time course of the peak currents (0 mV) before and after 3 μM nimodipine application. The application of nimodipine fully blocked the calcium currents, indicating the exclusive expression of L-type calcium currents. The L-type currents were obtained by the pharmacological subtraction of I-V currents’ family before and after nimodipine application (Figure 3B). The I-V relationship of L-type calcium currents was built using the pharmacological subtraction family and the biophysical properties were obtained by biophysical fit with the Boltzmann equation (Figure 3C). In these representative cells, activation threshold was around −30 mV with a peak current at 10 mV (V/2 and *k* of −12.0 and 5.6 mV respectively, estimated by fitting the experimental data with the Boltzmann equation). In line with previous reports, these biophysical properties fall between the embryonic and the adult isoform, possibly as a result of the simultaneous presence of both embryonic and adult splicing forms of Cav1.1 [49].

### 3.3. Resveratrol Affects the Biophysical Properties of Cav1.1 Currents

**Figure 4:**
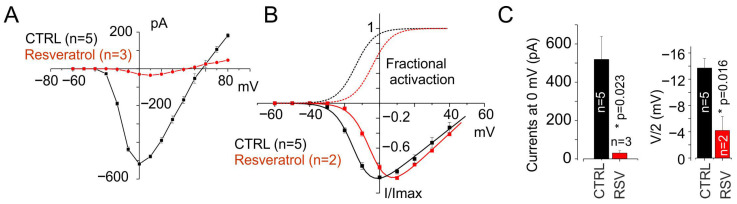
Resveratrol induces a loss of function in L-type calcium currents in C2C12 myocytes by altering their biophysical properties. (**A**) Mean current–voltage (I–V) relationship of nimodipine-sensitive calcium currents recorded from control (black trace, *n* = 5) and RES-pretreated (30 µM, 48 h, red trace, *n* = 3) C2C12 myocytes, under the same conditions described in Figure 3. Dots represent peak current values at the indicated voltage steps. (**B**) Normalized mean I–V relationships obtained from myocytes grown in the absence (CTRL, black trace, *n* = 5) or presence of RES (30 µM, 48 h, red trace, *n* = 2). Continuous lines show the best fits using the normalized Boltzmann equation: I_norm_= G_max(norm)_·(V − V_rev_)/(1 + exp[(V/2 − V)/k]). Fit parameters: for CTRL, V/2 = −13.7 mV, *k* = 5.4; for RES, V/2 = −4.2 mV, *k* = 5.5. Dashed lines represent the activation fraction derived from the same fits used for the normalized I–V data. (**C**) Bar plots showing the mean peak current at 0 mV (left) and the half-activation voltage (right) in control (black) and RES-treated cells (red).

Voltage-dependent calcium channels are essential for skeletal muscle function and undergo marked developmental changes. Specifically, in dystrophic conditions, myocytes often display embryonic-like calcium currents, as shown in Figure 3. RES, a polyphenol known to modulate gene expression and alternative splicing, has shown potential in modulating the expression of embryonic splice variants [38,39]. Previous studies have demonstrated its beneficial effects in various neuromuscular disorders, including DM1 [38,39]. Based on this background, we evaluated the impact of RES on VDCCs in a C2C12 myocyte model expressing embryonic-type calcium currents. A comparative study was performed to assess the pharmacological potential of RES in promoting the adult splice form current. We observed a loss of function, characterized by a strong reduction in calcium current amplitude across the entire voltage range explored (519 ± 120 and 30 ± 12 pA at 0 mV in CTRL and RES, respectively; *p* = 0.023; Figure 4A,C). The biophysical properties of the calcium current recorded from myocytes cultured with 30 µM RES showed a depolarizing shift in the half-activation voltage (V_1_/_2_) compared to control myocytes grown in the absence of the polyphenol (−13.7± 1.4 and −4.2 ± 2.1 in CTRL and RES, respectively; *p* = 0.016; Figure 4B,C).

**Figure 5:**
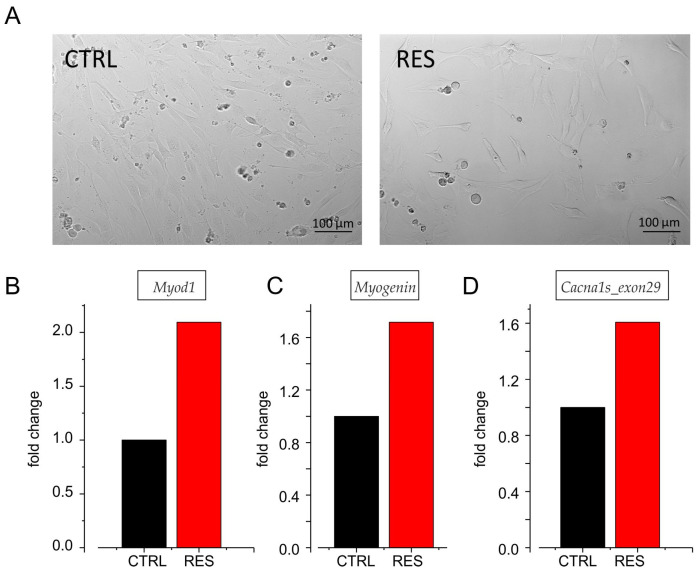
Resveratrol promotes myogenic differentiation and modulates the expression of Cav1.1 adult isoform transcripts in C2C12 cells and cardiac differentiation in CM-miPSCs. (**A**) Representative phase-contrast images of C2C12 cultures treated with vehicle (CTRL) or resveratrol (RES) for 72 h. RES-treated cultures display morphological features consistent with a more differentiated myogenic phenotype. (**B**,**C**) RT-qPCR analysis of the myogenic transcription factors *Myod1* (**B**) and *Myog* (**C**) in CTRL and RES-treated cells showing increased expression upon resveratrol treatment, consistent with activation of the myogenic program. (**D**) Expression analysis of *Cacna1s* transcripts containing exon 29 (adult Cav1.1 isoform) showing increased levels in RES-treated cells compared with controls. Data are expressed as fold change relative to CTRL.

Morphological changes associated with resveratrol treatment were first evaluated by phase-contrast microscopy. Representative images comparing CTRL- and RES-treated cultures at 72 h are shown in Figure 5A. Gene expression analysis revealed upregulation of the myogenic transcription factors *Myod1* (Figure 5B) and *Myog (*Figure 5C) in cells treated with resveratrol compared with vehicle-treated controls, indicating activation of the myogenic program. During the C2C12 myogenic program induced by resveratrol, we observed a clear upregulation of transcripts containing exon 29 (adult isoform) (Figure 5D), consistent with the electrophysiological effects reported in Figure 4A and with the observations reported by Tang et al. [32].

**Figure 6:**
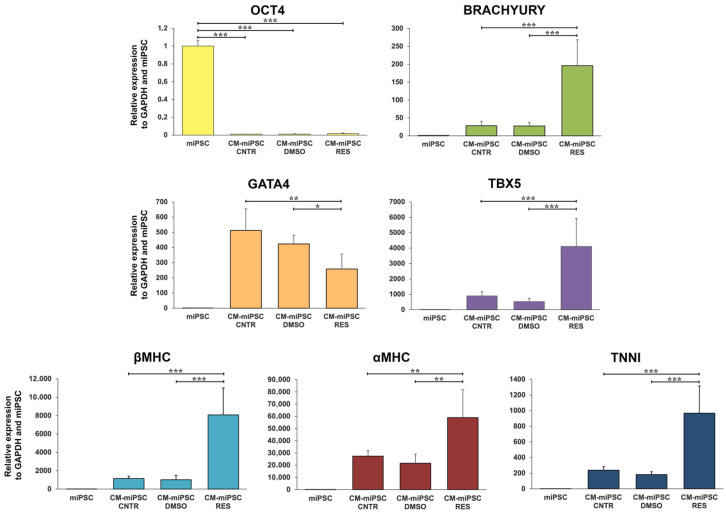
RES effects on the expression of cardiac differentiation markers in CM-miPSCs. qRT-PCR for the expression of genes related to pluripotency and to cardiac differentiation in CM-miPSCs treated with RES (CM-iPSCs RES). Controls are represented by undifferentiated iPSCs (miPSCs), non-treated CM-iPSCs (CM-iPSCs CNTR), and CM-iPSCs treated with DMSO (CM-iPSCs DMSO). Error bars represent ± SD. Student’s *t*-test, * *p* < 0.05, ** *p* < 0.01, *** *p* < 0.001.

Consistent with its pro-differentiative effects in myogenic lineages, we also observed a pro-differentiative effect of resveratrol in a well-established murine model of cardiomyogenesis generated by cellular reprogramming [46] (Figure 6). To evaluate the effect of resveratrol on cardiomyogenic differentiation, the expression of pluripotency and cardiac lineage markers was analyzed in miPSCs and cardiomyocyte-derived miPSCs (CM-miPSCs). Four experimental conditions were considered: (i) undifferentiated iPSCs, (ii) untreated CM-iPSCs (control), (iii) CM-iPSCs treated with DMSO (vehicle control), and (iv) CM-iPSCs treated with 15 μM RES. As expected, the pluripotency marker POU class 5 homeobox 1 (*OCT4*) was highly expressed in undifferentiated miPSCs, whereas its expression was markedly reduced in CM-miPSCs, confirming the loss of pluripotent identity during differentiation. In contrast, cardiogenic transcription factors, including *brachyury*, GATA binding protein 4 (*GATA4*), and T-box transcription factor 5 (*TBX5*), were significantly upregulated in CM-miPSCs compared with miPSCs, indicating the activation of early cardiac developmental programs. Notably, treatment with RES further enhanced the expression of these cardiogenic markers relative to control and DMSO-treated cells. A similar trend was observed for structural cardiac genes, including for the alpha chain of major histocompatibility complex (*αMHC*), *βMHC*, and troponin I (*TNNI*), which showed increased expression in RES-treated CM-miPSCs.

## 4. Discussion and Conclusions

The present study identifies a sequential pattern of ion channel expression during myogenic differentiation and demonstrates that RES modulates the biophysical properties of L-type calcium currents in C2C12 myocytes. These findings integrate electrophysiological and pharmacological observations into a coherent model of how membrane potential dynamics and calcium signaling coordinate muscle maturation, suggesting a potential therapeutic role for RES in muscular dystrophies characterized by abnormal calcium homeostasis and defective splicing, such as DM1. The progressive appearance of SK4, EAG, and Kir currents during C2C12 differentiation parallels the temporal sequence previously reported in human myoblasts [1,3]. Our data reveal that proliferating myoblasts express primarily SK4, maintaining a depolarized resting potential (~−20 mV). Upon induction of differentiation, SK4 currents decline and are replaced by EAG-like channels, followed by the appearance of Kir. This succession leads to a progressive hyperpolarization toward ~−70 mV, which is a key signal promoting calcium entry through VDCCs and the initiation of fusion. Such membrane potential remodeling is not merely a passive marker of differentiation, but an active regulator of gene expression and calcium signaling [2,41]. The shift in resting membrane potential facilitates the activation of LVA “window” calcium currents, which are essential for myoblast fusion in human cells [50]. Although C2C12 cells differ from primary human myoblasts in relying predominantly on L-type channels for calcium influx [48], the overall principle—progressive hyperpolarization enabling calcium signaling—remains conserved. RES exerts its action by reducing the peak amplitude of L-type calcium currents and inhibiting channel activation by shifting the steady-state activation curve rightward, in a concentration-dependent manner. While steady-state inactivation remains unaffected, it was also observed that RES significantly delays the channel recovery from inactivation [51].

Electrophysiological analysis of C2C12 myocytes confirmed that the predominant calcium influx occurs through L-type channels (Cav1.1), as nimodipine completely abolished inward currents. The observed activation threshold (~−30 mV) and half-activation voltage (V/2 ≈ −12 mV) fall between those typical of embryonic and adult isoforms of Cav1.1, suggesting co-expression of splice variants of *CACNA1S* [49]. Such hybrid activation profiles resemble those described in regenerating or dystrophic muscle fibers, where calcium handling reverts to an immature phenotype [31,32]. This intermediate gating behavior supports the hypothesis that C2C12 cells represent a valid model for studying the molecular mechanisms underlying the developmental switch in calcium channel isoforms. The persistence of embryonic-type channels in dystrophic conditions results in defective excitation–contraction coupling, prolonged calcium transients, and impaired contractility [32,52,53]. Therefore, compounds capable of promoting the adult Cav1.1 form may produce two main effects on the electrophysiological properties of Cav1.1 currents: (i) a strong reduction in calcium current amplitude, and (ii) a depolarizing shift in the activation curve (ΔV/2 ≈ +9 mV). Although this may appear as a “loss of function”, it is mechanistically consistent with a transition toward the adult isoform, which exhibits lower conductance and requires more positive potentials for activation [34]. The data indicates that RES promotes a maturation of calcium currents, rather than inhibition. RES is known to modulate gene expression and alternative splicing via multiple pathways, including activation of SIRT1 and AMPK, and inhibition of PDE4 [9,22]. Moreover, its ability to interact with nucleic acids [35] and influence chromatin structure may contribute to splicing regulation. In neuromuscular disease models, RES has corrected splicing abnormalities in *SMN2* [36,37], *GAA* [38], and *INSR* transcripts in DM1 fibroblasts [39]. Notably, Santoro et al. [38] showed that RES restored normal splicing of *RYR1* but not of *SERCA1*, and *CACNA1S* in DM1 myotubes. On the contrary, our transcriptomic analysis showed increased-mRNA-level expression of *CACNA1S* containing exon 29 after exposure to 5 µM RES for 72 h. Thus, the discrepancy in the results may be due to the exposure time (24 h vs. 72 h) to the polyphenol and to the treatment concentration (100 µM vs. 5 µM). Moreover, our findings extend these results to an electrophysiological level, showing that RES drives Cav1.1 behavior toward adult-like gating, consistent with the correction of splicing imbalance. The biophysical properties of the calcium current recorded from myocytes cultured with 30 µM RES showed a depolarizing shift in the V/2 compared to control myocytes grown in the absence of the polyphenol. Specifically, the V/2 shifted from −13.7 mV in control cells to −4.2 mV in RES-treated cells. This shift perfectly aligns the gating behavior of our treated cells with the established functional phenotype of fully mature skeletal muscle as demonstrated by Altomare et al. [24], who found that fully differentiated C2C12 myocytes expressing the skeletal Cav1.1 isoform exhibit a steady-state half-activation voltage of −3.5 ± 1.1 mV. Therefore, the RES-induced shift toward −4.2 mV indicates that the treated myocytes express Cav1.1 channel subunits typical of the mature adult isoform.

The coupling between potassium-driven hyperpolarization and calcium entry defines a functional axis in myogenesis. In early differentiation, depolarized SK4+ cells are fusion-incompetent. The emergence of EAG and Kir currents progressively hyperpolarizes the membrane, facilitating calcium influx through LVA or embryonic L-type channels and triggering fusion [2,51].

DM1 represents a paradigmatic example of a multisystemic disease caused by splicing dysregulation [32,52,53]. In skeletal muscle, mis-splicing of *CACNA1S* leads to persistent expression of embryonic Cav1.1 isoforms, contributing to calcium handling defects and muscle weakness. The ability of RES to modulate *CACNA1S* splicing and restore adult-type gating in our model suggests a potential therapeutic avenue. In addition to its splicing effects, RES exerts antioxidant, anti-inflammatory, and anti-fibrotic actions [13,14,23], which may further improve muscle integrity in dystrophic conditions. RES’ multi-target activity—combining transcriptional modulation, metabolic regulation, and ion channel modulation—makes it an attractive candidate for complex disorders like DM1. Its capacity to normalize electrophysiological function while enhancing mitochondrial metabolism [9] supports the concept of “metabolic reprogramming” as a therapeutic endpoint.

Collectively, our results suggest that RES influences myogenic differentiation through the coordinated modulation of potassium and calcium currents. It promotes the natural sequence of K^+^ current expression leading to hyperpolarization and leads to a functional shift in L-type calcium channels toward an adult phenotype. Our data also shows that resveratrol modulates the gene expression of pluripotency and cardiac lineage markers in miPSCs and cardiomyocyte-derived miPSCs, suggesting that RES promotes cardiomyogenic gene activation and may enhance the differentiation of CM-miPSCs toward a cardiac phenotype. This dual action implies that RES can act both as a myogenic enhancer and as a mender of electrophysiological immaturity, properties that align with its beneficial effects observed in various neuromuscular and cardiac models. Future studies should address the molecular mechanisms linking RES to *CACNA1S* splicing regulation, possibly through SIRT1- or AMPK-dependent pathways, and verify these effects in primary human myotubes or DM1 patient-derived cells. Integrating electrophysiological measurements with transcriptomic profiling will be crucial to confirm whether the biophysical remodeling we observed corresponds to specific splicing events. Moreover, long-term functional assays, including calcium imaging and contraction dynamics, will clarify the physiological relevance of the RES-induced Cav1.1 current modulation. In conclusion, this work highlights the importance of ion channel remodeling as both a marker and a driver of skeletal muscle differentiation. The electrophysiological signature induced by RES suggests that small molecules, capable of restoring adult calcium channel properties, could represent a promising pharmacological strategy for muscle regeneration and for the correction of splicing-related pathologies such as DM1.

## Data Availability

The original contributions presented in the study are included in the article. Further inquiries can be directed at the corresponding authors.
